# Formulation and In Vitro Evaluation of Oral Capsules from Liquid Herbal Antimalarials Marketed in Ghana

**DOI:** 10.1155/2021/6694664

**Published:** 2021-01-18

**Authors:** Christina Osei-Asare, Fredrick William Akuffo Owusu, Philomena Entsie, Ama Kwansima Annan, Rita Akosua Gyamaa, Edem Makafui Amenuke

**Affiliations:** ^1^Department of Pharmaceutics, School of Pharmacy, Central University, Miotso, Ghana; ^2^Department of Pharmaceutics, Faculty of Pharmacy and Pharmaceutical Sciences, Kwame Nkrumah University of Science and Technology, Kumasi, Ghana; ^3^Department of Pharmaceutical Sciences, Faculty of Health Sciences, Kumasi Technical University, Kumasi, Ghana; ^4^Department of Herbal Medicine, Faculty of Pharmacy and Pharmaceutical Sciences, Kwame Nkrumah University of Science and Technology, Kumasi, Ghana

## Abstract

Malaria ranks amongst the major health challenges faced by many developing countries. In Sub-Saharan and tropical regions of Africa, malaria continues to claim the life of one out of every twenty children below the age of five years. In adults, mortality rates are lower, but frequent debilitating attacks reduce the quality of life for chronic sufferers. The patronage and usage of liquid herbal antimalarials in the management and treatment of malaria in Ghana have been on the ascendency over the past decade. This project seeks to transform five liquid herbal antimalarial preparations (Agbeve pevah, Time mixture, Givers mixture, Masada mixture, and Rooter mixture) produced locally and commonly used for the treatment of malaria fever into capsules. This will help eliminate the current limitations, such as lack of patient compliance due to the bitterness and bulky nature of packaged preparation. The amount of dry extract per dose of each herbal antimalarial preparation and the wavelength of maximum absorption (*λ*_max_) of aqueous solutions of Agbeve, Time, Givers, Masada, and Rooter extract were determined. The flow properties of formulated granules were determined and subsequently encapsulated. The formulated capsules were evaluated using basic pharmacopeial tests, such as uniformity of weight, disintegration, drug content, and dissolution. Difference, *f*1, and similarity, *f*2, factors were employed in analyzing the dissolution profiles of the formulated capsules. The formulated granules exhibited good flow properties and passed the weight uniformity, disintegration, and drug content tests. The capsules exhibited optimal release of extract at the 45th minute in 0.1 M HCL. All formulated capsules had ƒ2 values >50 and ƒ1 values <15, indicating the similarity of their drug release profiles with their respective liquid herbal antimalarials. Oral capsules of Agbeve, Time, Givers, Masada, and Rooter have been successfully formulated and can be used as a substitute for Agbeve pevah, Time mixture, Givers mixture, Masada mixture, and Rooter mixture, respectively, in the treatment of malaria.

## 1. Introduction

According to the World Health Organization (WHO), malaria is a life-threatening disease caused by plasmodium parasites that are transmitted to people through the bites of infected female anopheles mosquitoes. Despite this prediction, it is noteworthy that malaria is a preventable and curable disease [1]. According to the newest world malaria report, released in December 2019, there were 228 million cases of malaria in 2018 compared with 231 million cases in 2017. The estimated number of malaria deaths stood at 405,000 in 2018, compared with 416,000 deaths in 2017. The WHO African region continues to carry a disproportionately high share of the global malaria burden. In 2018, the region accounted for 93% of malaria cases and 94% of malaria deaths [2]. This is as a result of *Plasmodium falciparum* being predominant in Africa. Moreover, the weather conditions in Africa allow for the transmission of the parasite, and the lack of resources and socioeconomic instability have hindered efficient malaria control activities [3]. Currently, the WHO recommends the use of either orthodox drugs or traditional (herbal) preparations in the prevention and treatment of malaria depending on the healthcare system and protocols in a country [1–3].

The use of plants for the purposes of healing predates civilization and is the basis of all modern medicines. Nonetheless, herbal products were eliminated from conventional medical use in the mid-20th century, not simply because they were ineffective but because they were not as economically profitable as the newer prescription (orthodox) medications [4–6]. However, herbal medicine is now the cornerstone of primary health care needs for about 75–80% of the world's population, especially in developing countries [7, 8]. This is largely due to the popular perception that natural medicines, besides being inexpensive and available locally, have little or no side effects. The use of natural medicines in the world, according to the World Health Organization (WHO), now exceeds by two to three times that of orthodox medications. Globally, over 1,200 plant species are known to be used for the treatment and management of malaria [7–9].

In Ghana, a number of strategies including the use of either an orthodox drug or a traditional (herbal) preparation are employed in the prevention and treatment of malaria. The orthodox medicines used mostly in malaria treatment are the artemisinin-based combination therapies (ACTs) [10, 11]. Even though ACTs are still efficient in the treatment and management of malaria, there is a strong upsurge in patronage of commercial herbal antimalarials in both rural and urban areas due to varied reasons such as accessibility, affordability, and efficacy [12–14]. These herbal antimalarial drugs which have been licensed by the Food and Drugs Authority (FDA) of Ghana for sale on the Ghanaian market usually consist of two or more extracts from plants and are mostly (˃90%) in liquid dosage forms [10, 11, 15].

These herbal liquid antimalarials may pose challenges such as unpleasant taste and smell which makes it difficult for patients to complete their medication regimen. Moreover, bulkiness of the liquid dosage form makes it inconvenient for patients to carry their medications along when going to their workplaces making them skip doses. Lack of measuring cups in the secondary and primary packaging of the medication leads to low accuracy of dosing or inconsistency. Herbal liquid preparations are also known to be more susceptible to microbial growth which can change the colour of the preparation and cause other serious infections [8, 16–19]. Based on the aforementioned challenges, this project sought to formulate a suitable solid dosage form (capsule), which will mask the bitter and unpleasant taste of these liquid herbal antimalarials, enhance the ease in handling the final product by patients, and ensure consistency in accurate dosing of five most commonly used liquid herbal antimalarial preparations on the Ghanaian market.

## 2. Materials and Methods

### 2.1. Materials

Agbeve pevah, Masada mixture, Time mixture, Givers mixture, and Rooter mixture were obtained from registered pharmacies in Accra, Ghana. Distilled water was obtained from the Department of Pharmaceutics, Central University, starch, Ernest Chemist Limited, Accra, Ghana, tragacanth gum and lactose from Sigma Aldrich, UK, and hard gelatin capsule shells from the Department of Pharmaceutics, KNUST. All other chemicals and reagents used in this study were of analytical grade.

### 2.2. Determining the Five Most Commonly Used Liquid Herbal Antimalarials

#### 2.2.1. Study Area

A random cross-sectional survey was conducted in the Ningo Prampram district in the Greater Accra region of Ghana.

#### 2.2.2. Period of Study

The study was conducted between September 2019 and November 2019.

#### 2.2.3. Questionnaire Pretesting

Prior to administration of the questionnaires, a pilot survey was carried out in the Ningo Prampram district to determine the suitability of the designed questionnaires. The consent of participants was sought prior to administration of questionnaires. Forty (40) questionnaires were issued to random respondents of both sexes to avoid bias. The questionnaires were read out to those who cannot read nor write. The suitability, competency, and duration of questionnaires were pretested and these produced Cronbach's alpha of 0.92, an indication that the questionnaires were well structured.

#### 2.2.4. Sample Size Selection

Using the population size of the district selected, 150 respondents were used for the survey [20].

#### 2.2.5. Sampling

Participants were randomly selected. The survey was an open gender-unbiased study. Individuals below the age of 12 years at the time of the questionnaire administration were excluded in the survey. Sampling at the street level was done randomly by interviewing every third individual or individuals from every pharmacy. At the community level, every second house was chosen, and the inhabitants were selected and interviewed.

#### 2.2.6. Data Collation and Analysis

Questionnaires were retrieved the same day from participants to avoid questionnaire loses. Questionnaires were collated and analyzed (frequency and percentage) using Statistical Package for the Social Sciences (SPSS).

### 2.3. Determination of Amount of Extract per Dose of the Herbal Antimalarials

According to the label instructions, the dose for Agbeve pevah, Masada mixture, Givers mixture, Time mixture, and Rooter are 45 mL, 30 mL, 60 mL, 45 mL, and 60 mL, respectively, which are all to be taken three times in a day. A dose of each drug was accurately measured and transferred into five crucible dishes, which had been previously weighed, cleaned, and dried. The preparations were evaporated to complete dryness at 60°C. The weight of each powdered extract was then recorded and used in subsequent calculations for encapsulation [21, 22].

### 2.4. Determination of Equivalent Amount of Extract in a Dose of Liquid and Dried Powder of the Sampled Herbal Antimalarials

One dose of the various sampled liquid herbal antimalarials was diluted with 0.1 M HCL and then scanned to obtain the maximum wavelength of absorption with corresponding absorbance. The dried powder from a dose of the various liquid herbal antimalarials was also diluted with 0.1 M HCL and then scanned to obtain the maximum wavelength of absorption with corresponding absorbance.

### 2.5. Preparation of Granules

Sixty doses of each herbal antimalarial were measured, transferred into different clean ceramic bowls, placed in an oven at 60°C, and evaporated to complete dryness. The dry extract equivalent to 50 doses of each herbal antimalarial was weighed and transferred into five different mortars, and its corresponding weight of starch (7.5% w/w) and lactose were then added by geometric dilution. The active ingredient and the excipient were blended to obtain a homogenous powder. Tragacanth mucilage (10% w/v) was added to the powder blend in each mortar to form a damp mass. The damp mass was sieved into granules using a sieve of aperture size 850 um. The damp granules were dried to obtain a constant weight. The dried granules were then screened using a 425 um sieve and stored in air tight containers pending further analysis.

### 2.6. Evaluation of the Flow Properties of Granules

A known weight of granules from each preparation was gently poured down the side of a 100 mL measuring cylinder and the initial fluff volume (*V*_o_) was noted. The measuring cylinder was then tapped 50 times and the final tapped volume (*V*_f_) was recorded. Carr's index and Hausner ratio were then calculated. The angle of repose of the granules was also determined using the fixed height method; a quantity of the granules was allowed to flow through a funnel clamped at a fixed height to a horizontal surface. The height and diameter of the resulting cone were measured and the angle of repose was calculated [23].

### 2.7. Formulation of Agbeve, Masada, Time, Givers, and Rooter Capsules

The dry Agbeve, Masada, Time, Givers, and Rooter granules were separately mixed with talc (1% w/w) and filled into hard gelatin capsule shells using a manual capsule filling machine. The capsules were prepared such that each capsule contained a dose of the liquid herbal antimalarial.

### 2.8. Determination of Equivalent Amount of Extract in a Dose of Dried Powder and a Formulated Capsule of the Sampled Herbal Antimalarials

One dose of the dried powder of the liquid herbal antimalarials was diluted with 0.1 M HCL and then scanned to obtain the maximum wavelength of absorption with corresponding absorbance. The dried granules from a dose (one capsule) of the formulated herbal antimalarial capsules was also diluted with 0.1 M HCL and then scanned to obtain the maximum wavelength of absorption with corresponding absorbance.

### 2.9. Assessment of Formulated Capsules

#### 2.9.1. Uniformity of Weight of Formulated Capsules

The weight of an intact filled capsule was determined using an analytical balance. The capsule was carefully opened making sure not to lose any shell material and the content was removed totally. The difference between the weight of the intact filled capsules and empty shell was calculated. The procedure was repeated for 19 more capsules. The mean weight of the 20 capsules was calculated and the percentage deviations from the mean were determined [24, 25].

#### 2.9.2. Disintegration Test of Formulated Capsules

The procedure in [24] was used. The bath was filled with water to the desired mark and the temperature was set at 37°C ± 0.5°C. The beaker was filled with 600 mL of distilled water and suspended in the main bath. The temperature was allowed to reach equilibrium with that of the bath. One capsule was put into each of the six tubes. A disc was placed on each capsule to prevent it from floating. A watch clock was set and the apparatus was operated until all six capsules had disintegrated leaving only remnants of gelatin shell on the mesh. The procedure was repeated twice for each formulation.

#### 2.9.3. Determination of Maximum Wavelength of Absorption of Extracts

One gram of the dried extract of Agbeve pevah was weighed and dissolved in a quantity of 0.1 M HCL in a 100 mL volumetric flask. 0.1 M HCL was added to the 100 mL mark and the solution was shaken. Serial dilutions of the solution were then made to obtain several concentrations. The solutions were scanned to obtain the maximum wavelength of absorption using a UV-Vis spectrophotometer. The same procedure was repeated for the dried extract of Masada, Time mixture, Givers mixture, and Rooter mixture, respectively.

#### 2.9.4. Calibration Curve for Agbeve, Masada, Time, Givers, and Rooter Dried Powder

Solutions of concentrations (0.06, 0.04, 0.03, 0.025, and 0.02% w/v; 0.03, 0.025, 0.02, and 0.01% w/v; 0.04, 0.03, 0.025, 0.02, and 0.01% w/v; 0.06, 0.04, 0.03, and 0.025% w/v; 0.04, 0.03, 0.025, 0.02, and 0.01% w/v) were prepared from the dried powders of Agbeve fever, Masada, Time mixture, Givers mixture, and Rooter mixture, respectively. The maximum wavelength of each extract was used in recording the corresponding absorbance. A calibration curve was then plotted for each product.

#### 2.9.5. Uniformity of Drug Content Test

The procedures described by [19, 22, 24] were modified and used. A dose (one capsule) was emptied into a beaker and dissolved in 100 mL of 0.1 M HCL. It was then filtered into a 100 mL volumetric flask and topped up with 0.1 M HCL to the 100 mL mark. A volume of 10 mL was drawn from the prepared solution and transferred into a different volumetric flask. It was topped up to the 100 mL mark with 0.1M HCL. The absorbance of the solution from each product was determined using a UV-Vis spectrophotometer at the maximum wavelength previously recorded. The procedure was repeated for nine other capsules from each formulated product.

#### 2.9.6. Dissolution Test for Formulated Capsules

The procedure described by [19, 22, 24] with slight modifications was used. The water bath was filled to the maximum mark. Nine hundred (900) mL of 0.1 MHCL was used in filling each of the six dissolution vessels in their respective compartments and they were held firmly in the bath. The thermostat was set at 37°C. The height of the basket apparatus was set at about 2 cm above the bottom of the beakers and the revolutions were set at 100 rpm. The dissolution medium was allowed to reach temperature equilibrium of 37°C ± 2°C. A formulated capsule of Agbeve was placed in the basket apparatus in each of the six vessels. At 5 min, 20 mL of the dissolution medium was withdrawn and filtered, after which 20 mL of the fresh medium was used to replace the withdrawn volume. The filtered 20 mL was serially diluted, and the absorbance recorded at the maximum wavelength previously determined. The absorbance was then fitted into the calibration equation to obtain the amount of extract released. The procedure was repeated at times 15, 30, 35, 40, 45, and 60 minutes. The data obtained was used in plotting a graph of cumulative drug released against time. The whole procedure was repeated for capsules of Masada, Time mixture, Givers mixture, and Rooter mixture.

#### 2.9.7. Dissolution Data Analysis

Model independent approach was used in investigating the difference (*f*1) and similarity factors (*f*2) between the formulated capsules and their reference liquid antimalarials [25].

## 3. Results and Discussion

### 3.1. Outcome of the Survey

**Figure 1:**
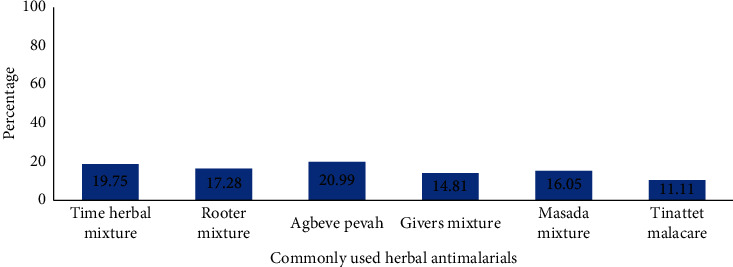
A bar chart showing the most commonly used liquid herbal antimalarials by respondents (*n* = 150) living within the Ningo Prampram district. The respondents indicated that Agbeve pevah was the herbal antimalarial drug they frequently used (20.99%), while Tinattet malacare was the least (11.11%) herbal antimalarial drug they used.

**Figure 2:**
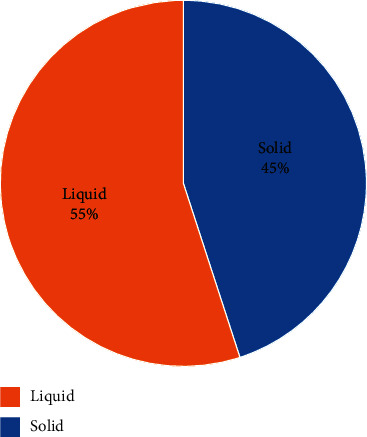
A pie chart showing respondents (*n* = 150) preference for liquid and solid dosage forms of herbal antimalarials in the Ningo Prampram district. 55% of the respondents in the Ningo Prampram district indicated that they preferred liquid dosage form of herbal antimalarials, while 45% indicated otherwise.

**Figure 3:**
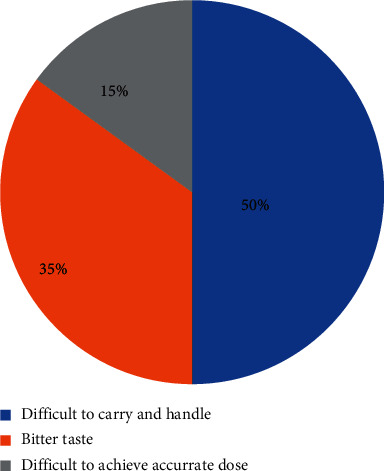
A pie chart showing reasons given by respondents (*n* = 150) for not preferring liquid preparations. 50% of the respondents in the Ningo Prampram district indicated that they had difficulty in handling and carrying the liquid preparations and hence they did not prefer this dosage form. 35% of the respondents also indicated that the bitter taste of the liquid preparations deterred them from choosing liquid preparations, while 15% indicated that they did not prefer the liquid preparations because of difficulty in obtaining an accurate dose.

The most common herbal antimalarial drug used by the respondents was Agbeve pevah, while Tinattet malacare was the least commonly used herbal antimalarial drug (Figure 1). It was also uncovered that the respondents preferred liquid dosage forms of herbal antimalarial drugs to solid dosage forms (Figure 2). This is due to the belief that the liquid dosage forms are used traditionally and hence the liquid dosage forms will be more effective than solid dosage forms. This may be a major reason why manufacturing companies of these herbal antimalarials are producing only the liquid dosage forms since it will appeal to the beliefs of the consumers and will increase patronage of their products. However, this belief is not true. Both liquid and solid dosage forms are of the same effectiveness once they have the same active ingredient in the same amount. Besides, it is evident from the reported literature [16, 19, 23] that solid dosage forms are more stable and aesthetically appealing and have accurate dosing and are less susceptible to microbial growth compared with liquid dosage forms. Thus, it will be of great benefit if consumers of herbal antimalarial preparations are educated on the efficacy of various dosage forms of these products. Stake holders such as Traditional and Alternative Medicines Directorate (TAMD), Ghana, Food and Drugs Authority (FDA), Ghana, and pharmaceutical manufacturing companies can spearhead education of consumers in this regard. Ultimately, the majority of the respondents also indicated that they do not prefer the liquid dosage form due to reasons such as bitter taste, bulky nature, and inaccurate dosing (Figure 3). This further corroborates the fact that conversion of these liquid antimalarials into capsules will enhance patient compliance to dosage regimen and further increase the patronage and usage of herbal antimalarial medications [16].

### 3.2. Equivalent Amount of Extract in a Dose of Liquid and Dried Powder of the Sampled Herbal Antimalarials

The dried powder of a dose for all the herbal antimalarials maintained the same maximum wavelength of absorption as the original liquid form with correspondingly similar absorbances (Table 1). All the sampled herbal antimalarials contained more than two plants with several active constituents. Thus, the results obtained in Table 1 indicate that the active constituents present in a dose of the liquid herbal antimalarials is the same in the dried extract which confirms that the method and temperature of drying did not result in a reduction in the active constituents present in the various herbal antimalarials [16, 26–29]. The dried powder of the various herbal antimalarials could therefore be subsequently used in formulating granules for encapsulation.

### 3.3. Flow Property of Granules

The flow behavior of granules can affect manufacturing efficiency and can directly affect product variables such as uniformity of content. The flow properties of Agbeve, Masada, Givers, Time, and Rooter granules were excellent according to Carr's index, Hausner ratio and angle of repose (Table 2). This excellent flow will enhance uniform filling of capsules during the encapsulation process [23].

### 3.4. Equivalent Amount of Extract in a Dose of Dried Powder and a Formulated Capsule of the Sampled Herbal Antimalarials

**Figure 4:**
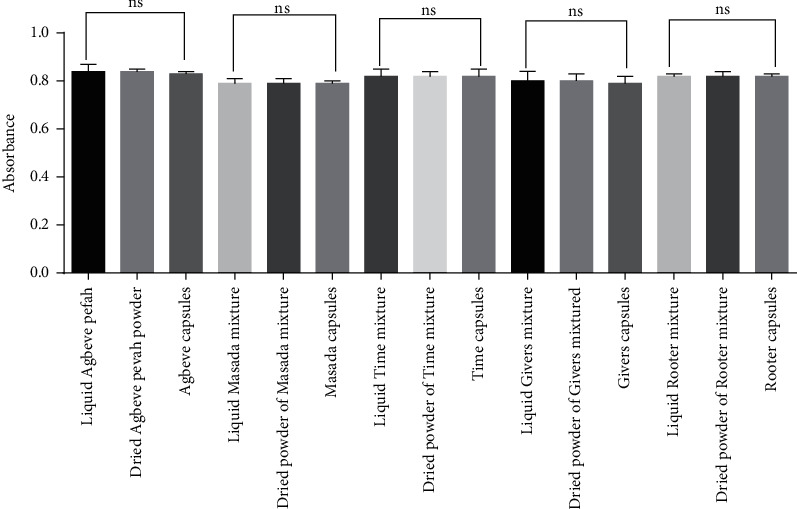
Statistical analysis on the absorbance of the extracts in the liquid herbal antimalarials, their dried powders, and formulated capsules (*n* = 3) using one-way ANOVA followed by Tukey's multiple comparisons test. *P* > 0.05; not significant (ns).

Analysis of granules in a formulated capsule (a dose) revealed that the extract in the capsule had the same maximum wavelength of absorption as the dried powder and original liquid form with similar absorbances (Table 3). Furthermore, statistical analysis on the absorbance from a dose of the liquid form, dried extract, and capsule of the various herbal antimalarials revealed no significant difference (*P* > 0.05) (Figure 4). This indicates that the amount of the active constituents in a dose of the liquid herbal antimalarial is the same as in the dried extract and is ultimately equal to that present in the capsules [16, 26–29]. As a result, the formulated capsule will produce the same effect as the liquid herbal antimalarial.

### 3.5. Quality Assessment of Formulated Capsules

#### 3.5.1. Uniformity of Weight

Uniformity of weight test ensures even distribution of ingredients in the dosage form. Uneven distribution may alter the dose in each individual drug and therefore may cause a lot of problems such as altering or affecting the therapeutic and toxic ranges of the drug. According to [18, 19], for capsules which are 250 mg or more, not more than two capsules should deviate from the average weight by ±5% and none should deviate by ±10%. On performing the uniformity of weight test on Agbeve, Masada, Rooter, Time, and Givers, it was found out that all capsules of the various drugs had weights well within the acceptable range (Table 4). This indicates that preencapsulation processes such as granulation and filling of the hard gelatin body were accurately and uniformly carried out [23].

#### 3.5.2. Disintegration Time of Formulated Capsules

Disintegration is the first step involved in a drug becoming bioavailable. It provides critical safety data on potential drug bioavailability in the body without having to utilize in vivo methods. Formulated capsules which fail the disintegration test may not release the drug promptly to provide the needed therapeutic effect. The disintegration time of hard gelatinous capsules should not exceed 30 min, according to [24]. All the formulated capsules passed this test (Table 5). This indicates that the prepared capsules will release the drug within the required time for dissolution to occur.

#### 3.5.3. Uniformity in Drug Content

Drug content uniformity is an important quality control test in the final assessment of a finished pharmaceutical product. It ensures that a consistent dose of the active ingredient is maintained between and amongst production batches so that the patient receives the right dose, avoiding underdosing and overdosing. Underdosing will produce suboptimal effects, while overdosing will produce undesirable adverse effects on the end user. As stated in [30], capsules pass this test if not more than one capsule lie outside the limits of 85% to 115%. The results in Table 6 show that the formulated Masada, Agbeve, Givers, Time, and Rooter capsules were within the limit, and this indicates that the extract contents of all the herbal antimalarial capsules are within specified acceptance limit and will not produce an underdosing or overdosing effect. Effective granulation, particle size distribution, and uniform filling of the capsule shells contributed to the consistency in drug contents of the formulated capsules.

#### 3.5.4. In Vitro Dissolution Studies

**Figure 5:**
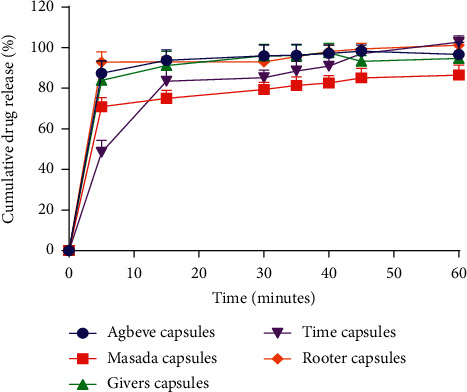
Dissolution profile of formulated Agbeve, Masada, Givers, Time, and Rooter capsules (*n* = 6).

Dissolution test measures the extent and rate of solution formation from a dosage form. Dissolution is important for drug bioavailability and therapeutic effectiveness [16, 19, 22, 23]. The British Pharmacopoeia stipulates that, for nonmodified release dosage forms, not less than 70% of the drug must be released by the 45th minute. All the formulated capsules passed the dissolution studies based on the British Pharmacopeia specifications (Figure 5). This means that the encapsulated extract can dissolve in physiological solution and be made available for absorption and subsequent pharmacological activity to occur. In effect, the formulated capsules will have the desired therapeutic action, and hence they are suitable for consumption by patients.

*(1) Dissolution Profile Comparison*. To ensure similarity in performance between a reference and formulated product, regulatory bodies are interested in knowing how similar the dissolution curves of the two products are, and this can be achieved by using the difference (*f*1) and similarity (*f*2) factors. The dissolution profiles of two products are found to be similar and considered bioequivalent, if *f*2 value is between 50 and 100 and *f*1 value is between 0 and 15 [31, 32]. All the formulated capsules had their *f*1 and *f*2 values falling within the standard range (Table 7), and therefore, their dissolution profile is similar to that of their reference liquid herbal antimalarial and hence they can be used interchangeably [32].

## 4. Conclusion

Agbeve, Masada, Givers, Time, and Rooter oral capsules were successfully developed from their various liquid preparations. The capsules passed all the pharmacopeial and nonpharmacopeial tests carried out on them. The oral capsules may therefore be used in place of liquid preparations for the treatment of malaria.

## Figures and Tables

**Table 1:** Maximum wavelength and corresponding absorbance for a dose of liquid and dried powder of the herbal antimalarials.

Sample used	Maximum wavelength of absorption (nm)	Absorbance
Liquid Agbeve pevah	300	0.84 ± 0.03
Dried Agbeve pevah powder	300	0.84 ± 0.01
Liquid Masada mixture	280	0.79 ± 0.02
Dried powder of Masada mixture	280	0.79 ± 0.02
Liquid Time mixture	280	0.82 ± 0.03
Dried powder of Time mixture	280	0.82 ± 0.02
Liquid Givers mixture	300	0.80 ± 0.04
Dried powder of Givers mixture	300	0.80 ± 0.03
Liquid Rooter mixture	320	0.82 ± 0.01
Dried powder of Rooter mixture	320	0.82 ± 0.02

**Table 2:** Flow properties of Agbeve, Masada, Givers, Time, and Rooter granules.

Formulated granules	Hausner's ratio	Carr's index %	Angle of repose
Agbeve fever	1.1	8.80	19.79
Masada mixture	1.1	9.61	19.44
Givers herbal mixture	1.05	4.99	19.90
Time mixture	1.04	4.60	20.00
Rooter mixture	1.09	8.31	18.92

**Table 3:** Maximum wavelength and corresponding absorbance for a dose of dried powder from the liquid herbal antimalaria and one capsule (dose) of the formulated herbal antimalarials.

Sample used	Maximum wavelength of absorption (nm)	Absorbance
Dried Agbeve pevah powder	300	0.84 ± 0.01
Agbeve capsule	300	0.83 ± 0.01
Dried powder of Masada mixture	280	0.79 ± 0.02
Masada capsule	280	0.79 ± 0.01
Dried powder of Time mixture	280	0.82 ± 0.02
Time capsule	280	0.82 ± 0.03
Dried powder of Givers mixture	300	0.80 ± 0.03
Givers capsule	300	0.79 ± 0.03
Dried powder of Rooter mixture	320	0.82 ± 0.02
Rooter capsule	320	0.82 ± 0.01

**Table 4:** Uniformity of weight of formulated capsules.

Formulated herbal antimalarial capsule	Average weight (mg)	Number of capsules deviating ±5%	Number of capsules deviating ±10%
Masada	255.4	Nil	Nil
Time	252.2	Nil	Nil
Rooter	253.5	Nil	Nil
Agbeve	250.0	Nil	Nil
Givers	254.0	Nil	Nil

**Table 5:** Disintegration time of the herbal antimalarial capsules.

Formulated herbal antimalarial capsule	Average disintegration time (minutes)
Agbeve fever	1.46 ± 0.54
Masada mixture	2.01 ± 0.89
Givers mixture	1.50 ± 0.76
Time mixture	1.25 ± 0.44
Rooter mixture	1.33 ± 0.63

**Table 6:** Drug content of formulated capsules.

Formulated herbal antimalarial capsule	Average drug content (%)
Agbeve fever	102.69 ± 0.854
Masaada mixture	97.88 ± 1.010
Givers mixture	102.31 ± 0.631
Time mixture	103.4 ± 0.931
Rooter mixture	101.4 ± 1.947

**Table 7:** Difference factor (*f*1) and similarity factor (*f*2) analysis between sampled liquid herbal antimalarials and their formulated capsules.

Formulation	Difference factor (*f*1)	Similarity factor (*f*2)	Comment
Agbeve capsule	5.01	74.44	Similar
Masada capsule	8.2	63.5	Similar
Time capsule	3.14	92.04	Similar
Givers capsule	5.26	72.77	Similar
Rooter capsule	7.01	67.34	Similar

## Data Availability

The data used to support the findings of this study are included in the article and are also available from the corresponding author upon request.
